# Serum Uric Acid is Associated with Renal Prognosis of Lupus Nephritis in Women but not in Men

**DOI:** 10.3390/jcm9030773

**Published:** 2020-03-12

**Authors:** Tae Ryom Oh, Hong Sang Choi, Chang Seong Kim, Dong-Ryeol Ryu, Sun-Hee Park, Shin Young Ahn, Soo Wan Kim, Eun Hui Bae, Seong Kwon Ma

**Affiliations:** 1 Department of Internal Medicine, Chonnam National University Medical School, Gwangju 61469, Korea; tryeomoh@daum.net (T.R.O.); hongsang38@hanmail.net (H.S.C.); laminion@daum.net (C.S.K.); skimw@chonnam.ac.kr (S.W.K.); 2 Department of Internal Medicine, College of Medicine, Ewha Womans University Seoul Hospital, Seoul 07985, Korea; drryu@ewha.ac.kr; 3 Division of Nephrology, Department of Internal Medicine, Kyungpook National University School of Medicine, Daegu 41944, Korea; sh-park@knu.ac.kr; 4 Department of Internal Medicine, Korea University College of Medicine, Seoul 02841, Korea; ahnshinyoung712@gmail.com

**Keywords:** lupus nephritis, hyperuricemia, uric acid, systemic lupus erythematosus, prognosis, proportional hazard models, end stage renal disease, sex

## Abstract

Lupus nephritis (LN) is a major complication of systemic lupus erythematosus. Early intervention in lupus nephritis improves prognosis. There is an association between hyperuricemia and lupus nephritis; nevertheless, the sex-specific role of uric acid in lupus nephritis remains unclear. We retrospectively analyzed 578 patients diagnosed with LN by renal biopsy. We determine the relationship of serum uric acid to progression of LN using Kaplan–Meier survival analyses and Cox proportional hazards models. The primary end point was LN progression defined as the initiation of dialysis or kidney transplantation. Men had higher mean serum uric acid levels than did women. Every 1 mg/dL increase in baseline uric acid level increased the risk of LN progression by 15.1%. The serum uric acid level was an independent risk factor for LN progression in women (hazard ratio [HR], 1.158; confidence interval [CI], 1.018–1.317; *p* = 0.028) but not in men (HR, 1.499; CI, 0.964–2.331; *p* = 0.072). Sensitivity analysis involving serum uric acid terciles generated consistent and robust results. Serum uric acid level was an independent risk factor for LN progression in women but not in men.

## 1. Introduction

Uric acid is the metabolite of purine that is excreted mainly in urine. The association between uric acid and various diseases has been reported. This relationship may be due to the antioxidant effect of uric acid [1] and its association with the immune system [2]. In diseases such as hypertension [3], cardiovascular disease [4], chronic kidney disease (CKD) [5], end-stage renal disease (ESRD) [6], and IgA nephropathy (IgAN) [7,8], uric acid is related to prognosis.

Lupus nephritis (LN) is a major complication of systemic lupus erythematosus (SLE), experienced by more than 50% of SLE patients [9]. If LN progresses to ESRD, there is a significant impact on quality of life [10] and socioeconomic status. Because early intervention on LN improves long-term survival and kidney damage [11], it is important to predict renal damage early. The prevalence of hyperuricemia in SLE patients is reported to be approximately 16.1% [12] and 29% [13]. In LN patients with CKD stages 1–3, the prevalence of HUA was 40.1% [14]. Despite the fact that hyperuricemia often occurs in SLE patients, the association between LN and hyperuricemia remains insufficiently studied. Furthermore, serum uric acid levels have been reported to have variable effects on chronic heart failure [15], cardiac hypertrophy in CKD patients [16], acute ischemic stroke [17] and IgAN [18], depending on sex; nevertheless, its sex-specific effect on LN remains unclear. In an analysis of the Korean National Health Insurance system, the prevalence of SLE in Korea was reported to be 18.8–21.7 per 100,000 people in 2006 and has been increasing since [19]. Despite this continued increase, there are few studies of the association between uric acid and renal survival in patients with lupus nephritis in Korea. Therefore, the purpose of this study was to determine the effect of uric acid on the long-term prognosis of lupus nephritis in Koreans and to identify variations in sex-specific influence.

## 2. Materials and Methods

### 2.1. Data Sources and Study Population

**Figure 1:**
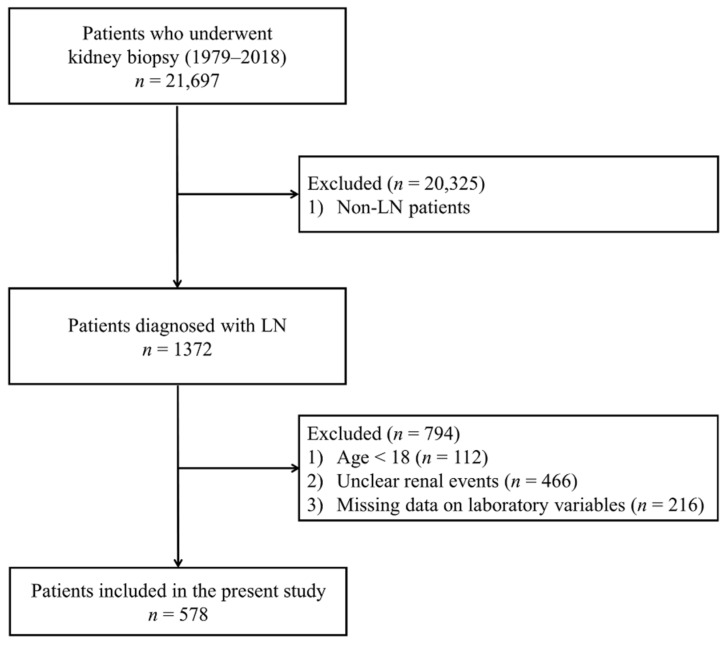
Flow diagram for patient’s enrollment. LN, Lupus nephritis.

Of the 21,697 patients who underwent kidney biopsies from January 1979 until October 2018 at 18 Korean university hospitals (Kyungpook National University Hospital, Kyung Hee University Hospital at Gandong, Kangdong Sacred Heart Hospital, Gangnam Severance Hospital, Korea University Guro Hospital, Korea University Anam Hospital, Eulji University Hospital, Seoul National University Boramae Medical Center, Seoul National University Bundang Hospital, Seoul National University Hospital, Severance Hospital, Pusan National University Yangsan Hospital, The Catholic University of Korea, Eunpyeong St. Mary’s Hospital, Ewha Womans University Mokdong Hospital, National Health Insurance Service Ilsan Hospital, Chonnam National University Hospital, Chonbuk National University Hospital and Hallym University Sacred Heart Hospital), 1372 patients were diagnosed with LN. We excluded 112 patients aged <18 years, 446 patients who did not know whether renal events had occurred, and 216 patients whose serum UA levels were not measured. Finally, the data from 578 patients were retrospectively analyzed (Figure 1).

### 2.2. Study End-Point, Definitions, and Measurements

The study’s primary end point was LN progression, defined as the initiation of dialysis or kidney transplantation. Anemia was defined as a hemoglobin level <13 g/dL in men and <12 g/dL in women [18]. The entire study population was classified into terciles based on the serum UA levels. The serum creatinine levels were measured using traceable isotope-dilution mass spectrometry. The eGFRs were calculated using the Modification of Diet in Renal Disease study equation [20].

### 2.3. Statistical Analyses

All continuous variables were tested for normality using the Shapiro–Wilk test. Normally distributed data are expressed as means and standard deviations (SDs), and skewed data are expressed as medians and interquartile ranges (IQR) (25^th^ percentile; 75^th^ percentile). To assess the clinical characteristics and differences between control group and hyperuricemia group, the Student’s t-test was used to assess the normally distributed variables, and the Mann–Whitney U test was used to analyze skewed data. Categorical variables are expressed as numbers and percentages, and the chi-squared test was used to compare groups. Except for total cholesterol (12.8%), anti-dsDNA antibody (22.0%), and urine protein creatinine ratio (UPCR) (29.4%), the percentage of missing values in all variables was less than 10%. Although the above three variables have a large proportion of missing data, Madley-Dowd et al. [20] reported that multiple imputation could reduce bias in missing random data. We used a multiple imputation method for missing data with ‘MICE’ package [21] in R because missing values in clinical data are mostly missing at random type [22]. The terciles of serum uric acid were used for sensitivity analyses. Kaplan–Meier survival curves with log-rank tests and a univariate Cox proportional hazards model were applied to evaluate the effect of the serum uric acid on LN progression. A multivariate Cox proportional hazards model was adjusted with the variables that may affect LN progression. Collinearity was analyzed to assess the variables’ interactions with the other independent variables. We used the Schoenfeld residuals test and log-minus-log survival plots to access the proportional hazards assumption of the Cox proportional hazards model. The hazard ratios (HRs) and 95% confidence intervals (CIs) were calculated to compare the risks of LN progression. We used restricted cubic spline curve to illustrate the non-linear association between the serum uric acid level and LN progression. The data were analyzed and plotted using R, version 3.5.2 [23]. All statistical tests were two-tailed, and a value of *p* < 0.05 was considered statistically significant.

### 2.4. Ethical Approval and Informed Consent

This study complied with the tenets of the Declaration of Helsinki. Because the database used in this study did not include personal identifiers and the study was retrospective and observational in its design, the need for informed consent was waived. The study was approved by Chonnam National University Hospital’s Institutional Review Board (CNUH-EXP-2019-369).

## 3. Results

### 3.1. Clinical Characteristics of the Study Population

**Figure 2:**
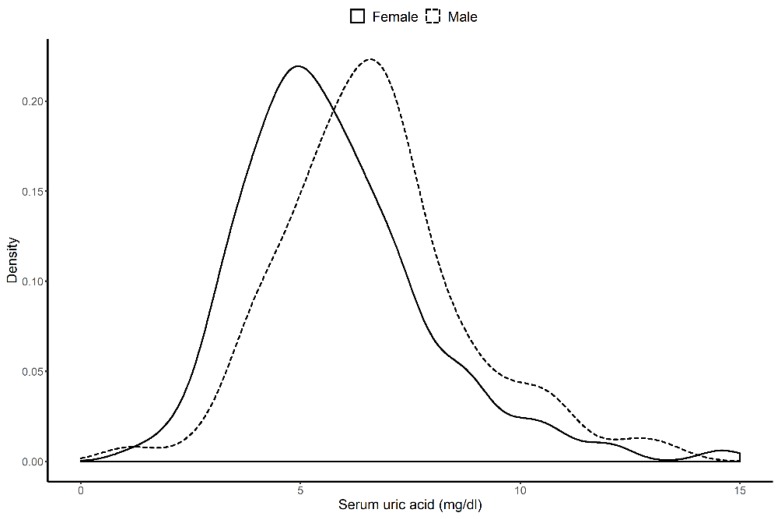
Difference in distribution of serum uric acid by sex. The median of serum uric acid level was 6.5 mg/dL in men and 5.4 mg/dL in women.

**Figure 3:**
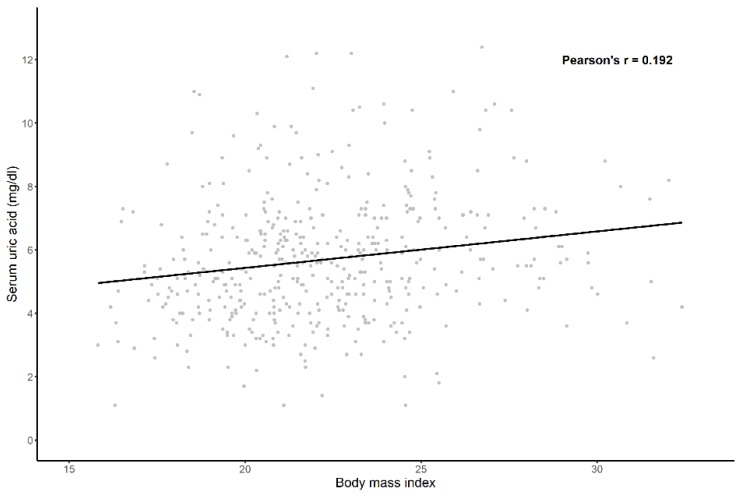
Association between body mass index and serum uric acid. Body mass index and serum uric acid showed positive correlation.

A total of 578 patients were analyzed. The median (IQR) follow-up duration was 6.72 years (3.03–10.65 years). The mean (±SD) age of all patients was 35.7 (±12.3) years, 86.0% of whom were women; 2.6% had diabetes mellitus (DM), and the median (IQR) of eGFR was 84.39 (59.47–111.62) mL/min/1.73 m^2^. The means (±SD) of systolic blood pressure and diastolic blood pressure were 123.62 (±17.54) and 77.42 (±11.66) mmHg, respectively. Based on the laboratory test at renal biopsy, 216 (37.3%) of the 578 patients had hyperuricemia. We summarize the difference of the clinical characteristics between control and hyperuricemia groups in Table 1. The hyperuricemia group showed no statistically significant difference in terms of prevalence of diabetes mellitus and hypertension compared with the control group. In addition, there was no significant difference in sex ratio or age between the groups. By contrast, creatinine levels were higher in the hyperuricemia group than in the control group. Figure 2 shows the significant difference in the distribution of the serum uric acid by sex. As the BMI increased, the serum uric acid level also tended to increase (Figure 3).

### 3.2. Crude Analysis of the Association between Serum Uric Acid Levels and Progression of Lupus Nephritis

**Figure 4:**
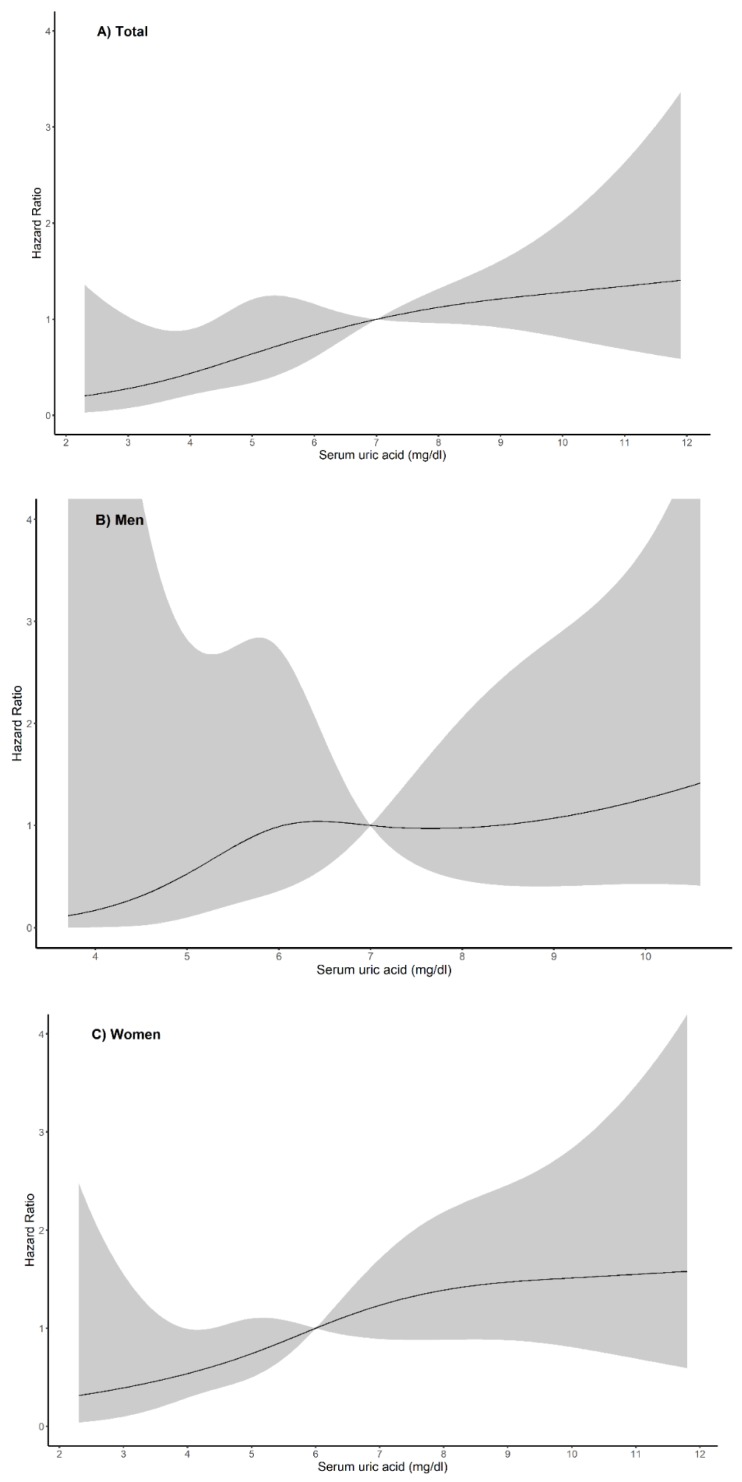
Restricted cubic spline curve of hazard ratio of serum uric for progression of lupus nephritis. Serum uric acid levels and progression of lupus nephritis showed a nonlinear relationship in total patients (**A**), in men (**B**), and in women (**C**).

During the follow-up period, a total of 51 (8.8%) patients experienced progression of LN. There were 24 (6.6%) LN progressions in the control group and 27 (12.5%) in the hyperuricemia group (*p* < 0.024). Among all patients, the serum uric acid level was associated with LN progression in crude analysis of Cox proportional hazard model (Table 2). However, the sex-specific Cox proportional model showed that the serum uric acid was only associated with progression of LN in women (HR, 1.150; 95% CI, 1.028–1.287; *p* = 0.015) not men (HR, 1.196; 95% CI, 0.914–1.565; *p* = 0.192). Figure 4 illustrates the non-linear relationships between the serum uric acid and LN progression in both sexes and within the entire study population.

### 3.3. Independent Risk Factors Associated with the Progression of Lupus Nephritis

**Figure 5:**
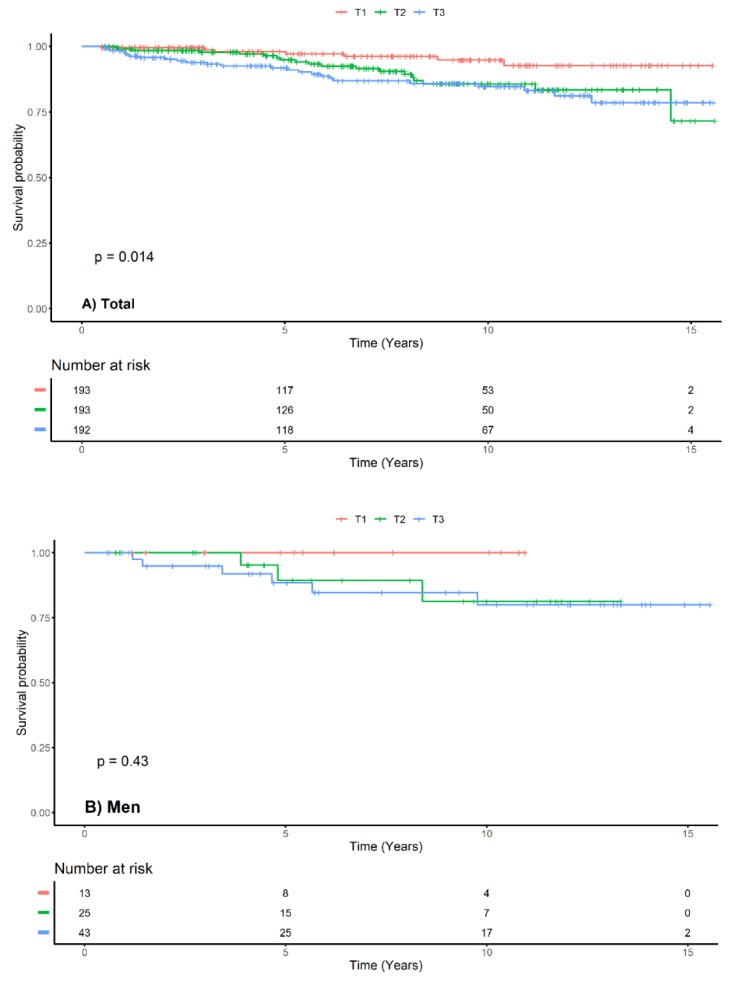

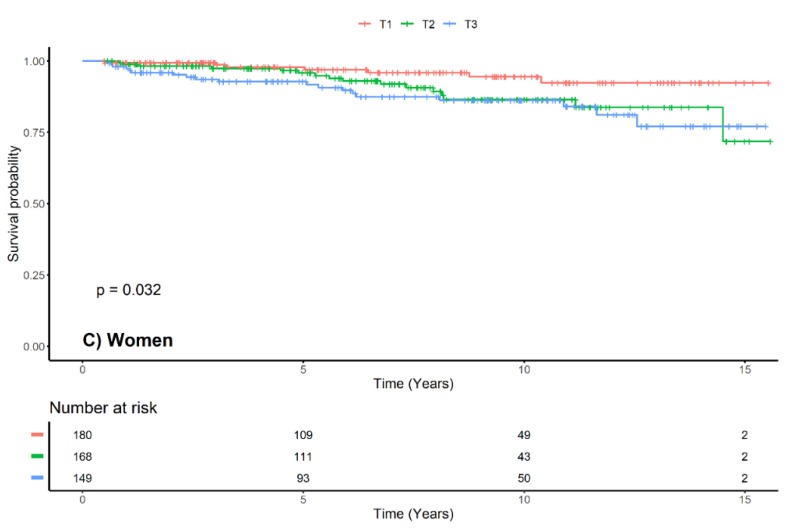
Kaplan–Meier survival curve with log-rank test between progression of LN and serum uric acid by sex. Kaplan–Meier survival curve for total patients (**A**), men (**B**) and women (**C**). Hyperuricemia was associated with progression of LN in total and women but not in men. The range of serum uric acid of terciles is 3.8 ± 0.8 mg/dL in T1, 5.6±0.5 mg/dL in T2 and 8.3 ± 1.9 mg/dL in T3 respectively. T1, tercile 1; T2, tercile 2; T3, tercile 3.

We used adjusted Cox proportional hazard models to determine the effect of the serum uric acid on progression of LN (Table 2). We used covariates for factors clinically known to be associated with the prognosis of LN and for variables with significant results in univariate analysis. We included the UPCR and anti-dsDNA antibody that had relatively large missing values in in the last Cox model (model 4). With the entire study population, the serum uric acid increased the risk of LN progression by nearly 15.1% for every 1 mg/dL raise in the baseline serum uric acid level (HR, 1.151; 95% CI, 1.025–1.292; *p =* 0.017). In the fully-adjusted Cox models, the serum uric acid showed an association with LN progression in women (HR, 1.158; 95% CI, 1.018–1.317; *p* < 0.028), but it did not show any significance for LN progression in men (HR, 1.499; 95% CI, 0.964–2.331; *p* < 0.072). We also analyzed the data using Kaplan–Meier survival analysis and log-rank tests on the terciles of the total study population and both sexes (Figure 5). Similar to Cox proportional hazard model analysis, the serum uric acid did not show any effect in men.

## 4. Discussion

Analyzing a total of 578 patients in this study, we found that the serum uric acid was an independent risk factor for LN progression. In addition, the serum uric acid level was associated with being women, not men.

Various biological mechanisms of serum uric acid have been reported that could explain their association with diseases. Uric acid is a powerful anti-oxidant that acts as a scavenger of singlet oxygen and radicals [1]. Uric acid aids in the maturation of dendritic cells and promotes the response of CD8 T cells when antigen is injected in vivo [2]. The importance of serum uric acid in neurologic diseases has been reported [24,25]. Uric acid has been reported to be a scavenger of per-oxynitrate in experimental allergic encephalomyelitis and multiple sclerosis [24]. In hyperglycemic mice, uric acid showed a protective effect in reducing the size of cerebral ischemia and reperfusion lesions. [25]. In vitro, mitochondria dysfunction was induced in hepatocytes exposed to uric acid, resulting in lipogenesis of hepatocytes and induction of fatty liver [26].

Various roles of uric acid have been identified in the kidney that could be associated with renal damage. In an animal model, uric acid was reported to induce vascular disease through a COX-2-dependent pathway [27]. In the cisplatin-induced AKI rat model, uric acid was shown to exacerbate renal injury even at concentrations that do not induce intrarenal crystal formation. [28]. Serum uric acid gave rise to renal damage in an acute kidney injury model through renal vasoconstriction (via activation of the renin-angiotensin system and reduction in NO of endothelial cell), antiangiogenic properties (via blockage of endothelial cell proliferation/migration, stimulation of endothelial cell apoptosis), proinflammatory properties (via activation of monocyte chemoattractant protein-1, C-reactive protein, NF-kB, and p38 mitogen-activated protein kinase) and alteration of renal autoregulation [29]. Interestingly, a study reported a link between uric acid and C3 in lupus nephritis. In vitro experiments showed that uric acid induces C3 activation [30]. The theoretical background may be that C3 activation by elevated serum uric acid may be associated with LN via classical and alternative pathways. Investigators also reported a negative correlation between C3 levels and uric acid in patients with LN [31].

The present study found that serum uric acid had different effects on renal prognosis depending on sex. The reason why serum uric acid has a sex-specific effect on diseases such as coronary artery disease [32,33], cardiac hypertrophy [16], IgA nephropathy [18], and others remains unclear. This phenomenon may be related to estrogen, which decreases the ability of urate transport 1 to lower serum uric acid levels and also promotes uric acid urine output [34]. Postmenopausal women have higher uric acid levels than premenopausal women, increasing the risk of atherosclerosis [35,36]. The use of women hormones in postmenopausal women reduced serum uric acid levels [37]. Furthermore, the sex-specific effect of uric acid may appear because women are more genetically susceptible to uric acid than men. For example, renal arteriolar hyalinosis occurred in women at lower serum uric acid concentrations than in men [38]. The glucose transporter 9 (GLUT9) encoded in the human SCL2A9 gene was also associated with serum uric acid levels; GLUT9 plays a major role in regulating serum uric acid concentrations [39,40]. It is interesting to note that more variations in GLUT9 were observed in women [39,41,42], and this may help explain the difference in susceptibility according to sex. This theoretical background may be the basis for the sex-specific effe ct of the uric acid. Nevertheless, substantial research is necessary to determine a clear causal relationship.

To the best of our knowledge, this is the first study to report the effect of serum uric acid on prognosis of LN that varies according to sex. It also represents the largest number of subjects analyzed in a single study with long follow-up. Although this study has many strengths, our analyses also have limitations. First, we did not determine a causal relationship between serum uric acid and renal prognosis in patients with LN. This is the limitation of all observational studies. Nevertheless, we used complementary analytical methods to investigate the relationship between the serum uric acid concentration and LN progression, because observational studies are useful tools for identifying epidemiologic associations [43]. Second, we used retrospective data. As a result, despite our efforts, there may be some overdiagnosis, underdiagnosis or misclassification of patients. Third, we could not consider hidden confounding factors and all the interrelationships between the variables in this study. There are factors that are not reflected because of limitations of the data (e.g., the effects of food on serum uric acid levels and disease activity of SLE). Fourth, there was a lack of data on the use of uric acid-lowering agents and subtypes of LN. The renal protective effects of these drugs have been studied recently; however, the data we analyzed have little information on them. Finally, we could not adjust our analysis for the subtype of LN because information on the subtype of LN, which is known as an important factor in the prognosis of LN, was not available.

## 5. Conclusions

We found that serum uric acid levels were an independent risk factor for LN progression in women not in men. Considering the existing literature and our findings, early detection and intervention of hyperuricemia in LN patients may improve renal prognosis.

## Figures and Tables

**Table 1:** Clinical characteristics of the subjects stratified by hyperuricemia.

Characteristics	All Subjects (*n* = 578)	Control (*n* = 362)	Hyperuricemia (*n* = 216)	*p*-Value
Age (year)	35.7 ± 12.3	35.58 ± 11.70	35.91 ± 13.15	0.786
Men (%)	81 (14.01%)	51 (14.09%)	30 (13.89%)	1.000
Height (cm)	160.00 [156.00;164.95]	160.00 [156.00;164.00]	160.00 [156.00;165.00]	0.640
Weight (cm)	57.00 [51.00;63.80]	56.00 [50.00;62.70]	58.00 [52.00;65.00]	0.005
Body mass index	21.88 [20.09;24.11]	21.64 [19.72;23.68]	22.48 [20.64;24.77]	0.001
Diabetes mellitus (%)	15 (2.60%)	8 (2.21%)	7 (3.24%)	0.629
Systolic blood pressure (mmHg)	123.62 ± 17.54	120.63 ± 16.43	128.64 ± 18.22	<0.001
Diastolic blood pressure (mmHg)	77.42 ± 11.66	75.46 ± 11.23	80.71 ± 11.65	<0.001
Serum uric acid (mg/dL)	5.90 ± 2.20	4.61 ± 1.10	8.06 ± 1.88	<0.001
Hemoglobin (g/dL)	10.53 ± 2.04	10.75 ± 1.90	10.15 ± 2.20	<0.001
Serum albumin (mg/dL)	2.89 ± 0.70	3.01 ± 0.68	2.69 ± 0.69	<0.001
C3	56.60 [37.00;80.30]	62.00 [42.10;85.40]	44.45 [31.90;69.65]	<0.001
Anti-dsDNA Ab (u/L)	33.15 [1.00;180.00]	23.20 [1.00;147.00]	50.25 [4.85;210.00]	0.014
Creatinine (mg/dL)	0.80 [0.60;1.10]	0.70 [0.59;0.89]	1.10 [0.80;1.48]	<0.001
eGFR (ml/min/1.73m^2^)	84.39 [59.47;111.62]	98.45 [76.41;123.37]	59.97 [43.03;84.30]	<0.001
Total cholesterol (mg/dL)	196.00 [161.00;236.00]	183.50 [155.00;222.00]	222.00 [178.00;267.50]	<0.001
Urine protein creatinine ratio (g/g Creatinine)	2.35 [1.03;4.73]	1.99 [0.80;3.85]	3.55 [1.73;6.69]	<0.001
Follow-up duration (year)	6.72 [3.03;10.65]	6.63 [2.98;10.35]	7.01 [3.26;10.96]	0.290

**Table 2:** Hazard ratio of serum uric acid for renal event with Cox proportional hazard models by sex.

	Total Subjects		Men		Women	
	HR [95% CI]	*p*-Value	HR [95% CI]	*p*-Value	HR [95% CI]	*p*-Value
Crude	1.156 [1.043;1.281]	0.006	1.196 [0.914;1.565]	0.192	1.150 [1.028;1.287]	0.015
Model 1	1.153 [1.040;1.279]	0.007	1.473 [1.058;2.052]	0.022	1.150 [1.027;1.286]	0.015
Model 2	1.154 [1.033;1.289]	0.012	1.410 [0.957;2.078]	0.082	1.151 [1.017;1.303]	0.026
Model 3	1.151 [1.025;1.292]	0.017	1.499 [0.964;2.331]	0.072	1.158 [1.018;1.317]	0.028

Model 1, crude + age, sex (exclude sex in subgroup analysis). Model 2, Model 1 + albumin, C3, creatinine, diabetes mellitus, systolic blood pressure and total cholesterol. Model 3, Model 2 + anti-dsDNA antibody and urine protein creatinine ratio. Abbreviation: CI, confidence interval; HR, hazard ratio.
